# COVID-19: Considerations for Medical Education during a Pandemic

**DOI:** 10.15694/mep.2020.000087.1

**Published:** 2020-05-01

**Authors:** Andjela Arandjelovic, Katarina Arandjelovic, Karen Dwyer, Cameron Shaw

**Affiliations:** 1 Deakin University; 2 Royal Melbourne Hospital

**Keywords:** Medical education, COVID-19, medical students during COVID-19, changes to medical schools during a pandemic, pandemic management education, medical students on the frontline

## Abstract

This article was migrated. The article was marked as recommended.

Clinical placement has been the cornerstone of medical training since the early foundations of the medical profession. The COVID-19 pandemic generates unprecedented challenges for the delivery of medical education, particularly in the setting of ‘flatten the curve’ public health initiatives to curtail transmission. As the number of cases of COVID-19 increase, hospitals are limiting medical students’ attendance at ward rounds, clinics and theatre, representing a fundamental shift in clinical education from the bedside to online formats.

We discuss the considerations behind these changes, review the strategies implemented during previous global infectious disease epidemics, and suggest strategies for maximising clinical education going forward.

## Introduction

In December 2019, a novel coronavirus (SARS-CoV-2) emerged from the central-Chinese city of Wuhan and rapidly evolved into a global pandemic ( Australian Government Department of Health, 2020). Initiatives designed to curtail transmission, or *flatten the curve*, have posed challenges for the delivery of medical education. The response of medical schools and partnering health services has been heterogenous. Recognising an important learning opportunity, some services have opted for a business-as-usual approach, while others have limited students’ attendance at ward rounds and clinics. A number have transitioned to wholly-online teaching, in line with principles of physical distancing ( Kelso, Milne and Kelly, 2009). Whilst there is need to adapt the medical curriculum to facilitate timely graduation of medical students, this situation also presents an opportunity to incorporate innovative teaching and assessment methods to meet future disruptions.

## Pandemic Management in Future Medical Practice

Climate change-induced extreme weather events and global warming will increase the frequency and severity of infectious disease outbreaks ( Watts *et al.*, 2018). Pandemic management, therefore, will constitute an important part of future medical practice. Assessment of medical students’ preparedness for a H5N1 pandemic demonstrated insufficient knowledge of disease pathophysiology, with over half the students obtaining their knowledge from media sources, rather than university material ( Herman *et al.*, 2007; Mortelmans *et al.*, 2009). To better prepare future doctors for future pandemics, it is essential to include pandemic-related content in the medical curriculum. This may consist of simulated environments, which have proven to be an effective component of pandemic-management training, both for technical and nontechnical skills ( Elcin *et al.*, 2016).

## Considerations for Patient Contact-Based Teaching

Continuing ward-based teaching must prioritise safety. The donning and doffing of Personal Protective Equipment (PPE) represents a risk of contact with contaminated fluids, and thus disease transmission. Incorrect PPE doffing has been implicated in a cluster of SARS cases among healthcare staff in Canada ( Centers for Disease Control and Prevention (CDC), 2003). It is imperative that medical students have access to and be trained in the use of PPE regardless of duty, given the prolonged aerosol and surface stability of SARS-CoV-2 ( van Doremalen *et al.*, 2020). There are already widespread shortages in PPE, particularly N95 masks, and the presence of medical students will increase the number of PPE used per clinical encounter ( Murphy, 2020). Should medical students be invited to continue clinical placement, or to participate in the COVID-19 workforce, this increase in demand ought to be accounted for.

It is recognised more broadly that medical students receive inadequate PPE training. An audit of US medical students found that 59% of participants had not received any PPE training, and during simulations only 7% exhibited correct donning and doffing technique, highlighting the need to both teach and reinforce ( John *et al*., 2017). The emphasis on hand hygiene among medical students during the SARS outbreak resulted in a significantly higher compliance the following year ( Wong and Tam, 2005). Investing in PPE training in the current climate will likely have ongoing benefits.

## Considerations for Non-Patient Contact-Based Teaching

Should the need arise to cease all face-to-face teaching and clinical placements during a pandemic, online-based teaching methods must be accessible to medical students without delay. Universities globally are already transitioning to online teaching formats. Remote access to live tutorials and problem-based learning is possible through the use of video conferencing applications such as Zoom. During the SARS outbreak, universities in Singapore and Canada incorporated videotaped vignettes and mannequin simulators as patient surrogates ( Lim *et al*., 2009; Park *et al*., 2016). As a substitute for anatomy laboratories, a US university is providing online labelled images of cadavers and pathology specimen pots ( University of Michigan Medical School, 2020).

There has also been increasing use of Telehealth. In Australia, government subsidies have facilitated the expansion of existing services, in order to support physical distancing measures ( Royal Australian College of General Practitioners, 2020). Telehealth offers an avenue for the continuation of parallel consulting by physicians and medical students. This may preserve the development of interpersonal skills, which may otherwise be difficult to attain solely through online or simulated learning.

## Considerations for Conducting University Assessments

The conduct of assessments must also be adapted. The Imperial College London has successfully conducted final year exams online, representing a global first ( Tapper, Batty and Savage, 2020). Deakin University has also successfully conducted online examinations for preclinical students through Practique, and The University of Queensland has announced they will be conducting examinations online via Proctortrack. This is an emerging concept as both medical school and specialty training written examinations are traditionally paper-based, and we welcome novel approaches to assessments going forward.

## Workforce Strategies

Medical students may represent a valuable surge mechanism in times of heightened demand on the medical workforce. The British General Medical Council (GMC) has offered ultimate-year medical students the opportunity to graduate early and be absorbed into the workforce, on the proviso that they have met requirements for provisional registration ( General Medical Council, 2020). An Australian health service has developed the role of ‘clinical assistant’ whereby medical students will provide clinical support roles that may be administrative in nature, or clinically supportive, such as phlebotomy ( Western Health, 2020).

The organisation representing the Medical Deans of Australia and New Zealand (MDANZ) provides a good framework for the recruitment of medical students into such roles, and these principles are demonstrated in table 1. We advocate for the consideration of these principles when devising such roles ( Craig, 2020).

**Table 1.** Principles for the participation of medical students in the COVID-19 response

**1. Choice** • Participation in the COVID-19 response must be voluntary and free from coercion • While this may complement course material, it is not considered part of the curriculum, and students will not be disadvantaged with regards to course work if they decide against volunteering • Students who choose not to participate in this work are offered alternate learning pathways
**2. Safety** • Appropriate use access to and effective training in the use of PPE • Additional precautions for students with pre-existing health risks, e.g. immunocompromise
**3. Role Clarity** • Identification of the scope of practice (including limitations) to the student and the broader team
**4. Indemnity** • All medical students must have full indemnity insurance for the designated tasks
**5. Competency** • Tasks delegated to students must be at their level of competence • Where the tasks are beyond their training, additional training must be provided
**6. Supervision and Support** • Adequate supervision must be provided at all times, including emotional supports • Designated tasks should prioritise the education of medical students alongside service provision • Pathways for the escalation of concerns by medical students must be available, ideally independent from the health service
**7. Remuneration** • Service provision roles must be appropriately remunerated
**8. Accountability** • The university remains accountable for the learning, physical and emotional wellbeing of its medical students participating in the COVID-19 response

## Ethical Considerations

The involvement of medical students in the COVID-19 workforce must be thoroughly scrutinised from an ethical perspective. While there is valuable experience to be gained from this opportunity, the risk of harm to patients in inexperienced and possibly under-supervised hands challenges the principles of non-maleficence ( Mortelmans *et al.*, 2009). Conversely, medical student participation in the COVID-19 response will develop a cohort of pandemic-cogent practitioners, and will inform and inspire their future careers. Redirecting tasks to medical students, while reducing the burden on the existing workforce, represents improved use of resources and staff.

Tasks delegated to students ought to depend on stage of training along with confidence and willingness to participate. It must be remembered that while hospital staff have professional and contractual obligations to their workplace, medical students do not - posing medicolegal and industrial relations considerations ( Lim *et al.*, 2009). Should medical students be involved in the COVID-19 response, we encourage a formalised contract with the health service provider and renumeration.

Finally, all medical students must ultimately meet competencies for graduation as designated by the relevant national governance body. The participation of medical students in the pandemic effort must not influence their progress through medical school, thereby safeguarding the voluntary nature of this endeavour.

## Conclusion

The COVID-19 pandemic represents a disruption to medical education, and has demonstrated an urgent need for innovation. Recognising the importance of clinical placement in the medical curriculum, the requirement for physical distancing during a pandemic has led to greater uptake of online-based teaching and telehealth services. There may be a role for medical students to voluntarily contribute to the COVID-19 response, however this must be guided by frameworks prioritising safety. Ultimately, adapting the medical curriculum to involve pandemic management training, will lead to a generation of graduates prepared to respond to future global infectious disease outbreaks.

## Take Home Messages

• The COVID-19 pandemic generates unprecedented challenges for the delivery of education to medical students
• This represents a need for innovative teaching and assessment methods to prevent disruption to course progession
• If medical students were to be recruited on the frontline, or patient contact-based teaching was to continue, adequate PPE and training must be provided
• Non-patient contact-based teaching may involve video conferencing applications and telehealth
• Ultimately, COVID-19 has demonstrated the need to adapt the medical curriculum to involve pandemic management training, so as to develop a generation of graduates prepared to respond to future global infectious disease outbreaks

## Notes On Contributors

**Ms Andjela Arandjelovic**: a third year Doctor of Medicine student at Deakin University, Australia. ORCID iD: https://orcid.org/0000-0002-8145-1086

**Dr Katarina Arandjelovic**: an anasthetic registrar at the Royal Melbourne Hospital, Australia.

**Prof Karen Dwyer**: Dean of Medicine at Deakin University, and a Nephrologist and Transplant Physician at the University Hospital Geelong and Epworth Geelong.

**A/Prof Cameron Shaw**: Director of Clinical Studies at Geelong Clinical School, Deakin University. Also a Neurologist at University Hospital Geelong and Epworth Geelong.
