# Supplementary material for: COVID-19: Considerations for Medical Education during a Pandemic
Source: MedEdPublish (2016). 2020 May 1;9:87. [Version 1] doi: 10.15694/mep.2020.000087.1 (PMC10697469; doi:10.15694/mep.2020.000087.1)
Supplement: Peer review history

# Reviewer report: Reviewer response for version 1

**DOI:** 10.21956/mep.20074.r30879

**Published:** 2020-05-24

**Authors:** Leila Niemi-Murola

**Affiliations:** 1 University of Helsinki

This review has been migrated. The reviewer awarded 4 stars out of 5 This is an interesting article focusing on the challenges brought up by the recent pandemic. Personnel working in wards and emergencies have been too involved in patient care to teach and there have been concerns for the students’ safety, too. Teachers have successfully solved many problems by constructing innovative e-learning, but the challenges related to hands-on teaching remain. As the authors point out, there will be new pandemics in the future and we should give them preparedness to face challenges related to them.As the authors point out, patient contact-based teaching will be a problem if there is a shortage of protective clothing or difficulties in maintaining proper hand hygiene. Teleconferences and simulations have been implemented successfully in many places. The pandemic has also encouraged universities to facilitate use of electronic assessment, too. As the authors emphasize, there are several ethical considerations when planning teaching and learning for and during a pandemic.The authors give a valuable summary of their principles in the Table 1.

Reviewer Expertise:

NA

No decision status is available

# Reviewer report: Reviewer response for version 1

**DOI:** 10.21956/mep.20074.r30884

**Published:** 2020-05-04

**Authors:** P Ravi Shankar

**Affiliations:** 1 American International Medical University

This review has been migrated. The reviewer awarded 4 stars out of 5 This is another in a series of manuscripts about COVID-19 and medical education. The authors briefly examine many areas and the possible impact of the pandemic on these areas. The topic for pandemic preparedness is important and can be offered in an integrated manner with other health science and broader disciplines. The major hurdle remains direct patient contact and the learning of physical examination skills. Many other skills like interviewing can be learned online. Assessment of physical examination skills will be another challenge as it will be difficult to be carried out remotely. The involvement of medical students in the health workforce and ethical considerations regarding the same have been well addressed. This is an interesting addition to the growing literature on COVID-19 and health professions education.

Reviewer Expertise:

NA

No decision status is available

# Reviewer report: Reviewer response for version 1

**DOI:** 10.21956/mep.20074.r30883

**Published:** 2020-05-03

**Authors:** Balakrishnan Nair

**Affiliations:** 1 Centre for Medical Professional Development and University of Newcastle

This review has been migrated. The reviewer awarded 3 stars out of 5 I enjoyed reading this article. In this challenging period in medical education, any suggestions will be welcome. I found the table to be very useful and keep this a checklist. There are many learning points in the current situation. We can teach the students about telehealth consultations, their limitations , management of crises in healthcare and professionalism. These are valuable skills for the future . Assessment is a major challenge now and we have to be innovative .One has to look for more reliable and valid tools and improvise as some other recent articles suggested.

Reviewer Expertise:

NA

No decision status is available

# Reviewer report: Reviewer response for version 1

**DOI:** 10.21956/mep.20074.r30882

**Published:** 2020-05-03

**Authors:** Mohamed Hassan Taha

**Affiliations:** 1 College of Medicine, Univeristy of Sharjah

This review has been migrated. The reviewer awarded 4 stars out of 5 An interesting paper, the authors provided a comprehensive layout of various aspects of medical education for Clinical placement During COVID-19. The authors discussed these considerations with a critical review of the strategies implemented during previous global infectious disease epidemics Such as SARS. This work is a nucleus for further researches.

Reviewer Expertise:

NA

No decision status is available

# Reviewer report: Reviewer response for version 1

**DOI:** 10.21956/mep.20074.r30881

**Published:** 2020-05-02

**Authors:** Trevor Gibbs

**Affiliations:** 1 AMEE

This review has been migrated. The reviewer awarded 4 stars out of 5 An interesting paper to read which has brought to the attention of the reader some points worthy of discussion. The paper encapsulated many of our own thoughts, specifically regarding the future, which of course is equally important as the present.I was looking to read more about the consequences of the changes that we need to make , both in the educational arena and in the effects upon student and faculty wellbeing.I do feel however that it adds enough to the discussion to recommend that it is read by all faculty and the Table 1, I recommend to all faculty and students.

Reviewer Expertise:

NA

No decision status is available

# Reviewer report: Reviewer response for version 1

**DOI:** 10.21956/mep.20074.r30880

**Published:** 2020-05-01

**Authors:** Dujeepa D. Samarasekera

**Affiliations:** 1 National University of Singapore

This review has been migrated. The reviewer awarded 3 stars out of 5 Interesting and useful discussion. The authors share a few important considerations to focus during the current crisis and also for any future epidemics or pandemics. I would have preferred if the authors have also highlighted any challenges or limitations when following these guidelines or considerations. Furthermore, areas such as student and faculty wellbeing and how best to mitigate longterm effects should be included.

Reviewer Expertise:

NA

No decision status is available

# Reviewer report: Reviewer response for version 1

**DOI:** 10.21956/mep.20074.r30878

**Published:** 2020-05-01

**Authors:** Daniel Xu

**Affiliations:** 1 Curtin Medical School

This review has been migrated. The reviewer awarded 3 stars out of 5 An comprehensive layout of various aspects of medical education, it'll be better that detailed solutions can be provided in delivering the curriculum in clinical placement. We understand the limitation in the Australian setting.

Reviewer Expertise:

NA

No decision status is available
